# Health Care Resource Utilization in Adults Living With Type 1 Diabetes Mellitus in the South African Public Health Sector: Protocol for a 1-Year Retrospective Analysis With a 5-, 10-, and 25-Year Projection

**DOI:** 10.2196/44308

**Published:** 2023-02-13

**Authors:** Sindeep Bhana, Poobalan Naidoo, Somasundram Pillay, Ebrahim Variava, Kiolan Naidoo, Neeresh Rohitlall, Lauren Sekhuthe, Bruno Pauly

**Affiliations:** 1 Chris Hani Baragwanath Hospital Complex Johannesburg South Africa; 2 King Edward VIII Hospital Durban South Africa; 3 Klerksdorp Tshepong Hospital Complex Klerksdorp South Africa; 4 Sanofi Johannesburg South Africa

**Keywords:** type 1 diabetes mellitus, South African public health care system, health care resource utilization, pharmacoeconomics, diabetes, adults, insulin, essential, policy makers

## Abstract

**Background:**

Type 1 diabetes mellitus (T1DM) is less common than type 2 diabetes mellitus but is increasing in frequency in South Africa. It tends to affect younger individuals, and upon diagnosis, exogenous insulin is essential for survival. In South Africa, the health care system is divided into private and public health care systems. The private system is well resourced, whereas the public sector, which treats more than 80% of the population, has minimal resources. There are currently no studies in South Africa, and Africa at large, that have evaluated the immediate and long-term costs of managing people living with T1DM in the public sector.

**Objective:**

The primary objective was to quantify the cost of health care resource utilization over a 12-month period in patients with controlled and uncontrolled T1DM in the public health care sector. In addition, we will project costs for 5, 10, and 25 years and determine if there are cost differences in managing subsets of patients who achieve glycemic control (hemoglobin A_1c_ [HbA_1c_] <7%) and those who do not.

**Methods:**

The study was performed in accordance with Good Epidemiological Practice. Ethical clearance and institutional permissions were acquired. Clinical data were collected from 2 tertiary hospitals in South Africa. Patients with T1DM, who provided written informed consent, and who satisfied the inclusion criteria were enrolled in the study. Data collection included demographic and clinical characteristics, acute and chronic complications, hospital admissions, and so on. We plan to perform a cost-effectiveness analysis to quantify the costs of health care utilization in the preceding 12 months. In addition, we will estimate projected costs over the next 10 years, assuming that study participants maintain their current HbA_1c_ level. The cost-effectiveness analysis will be modeled using the IQVIA CORE Diabetes Model. The primary outcome measures are incremental quality-adjusted life years, incremental costs, incremental cost-effectiveness ratios, and incremental life years.

**Results:**

Ethical clearance and institutional approval were obtained (reference number 200407). Enrollment began on February 9, 2021, and was completed on August 24, 2021, with 224 participants. A database lock was performed on October 29, 2021. The statistical analysis and clinical study report were completed in January 2022.

**Conclusions:**

At present, there are no data assessing the short- and long-term costs of managing patients with T1DM in the South African public sector. It is hoped that the findings of this study will help policy makers optimally use limited resources to reduce morbidity and mortality in people living with T1DM.

**International Registered Report Identifier (IRRID):**

RR1-10.2196/44308

## Introduction

### Background

Approximately 11.3% of the South African population is living with diabetes [1], and according to a Statistics South Africa report on mortality and causes of death released in 2020, diabetes is the second most common cause of death in the country [2]. Although type 2 diabetes mellitus (T2DM) is more prevalent than type 1 diabetes mellitus (T1DM), the incidence of T1DM is increasing annually at rates of 2% to 5% [1].

T1DM is an autoimmune disease typically occurring in childhood or early adulthood, resulting in the inability to produce enough insulin because of the destruction of insulin-producing islet cells in the pancreas [2]. It is a debilitating disease with life-threatening acute and chronic complications such as diabetic ketoacidosis, coronary heart disease, cerebrovascular disease, retinopathy, and nephropathy. The financial burden associated with the management of this disease is significant [3]. Early diagnosis and intervention can improve morbidity and mortality. Despite improving globally, diabetes management in developing countries is lagging, predisposing people living with diabetes to increased morbidity and mortality [4].

Health care in South Africa is characterized by 2 disparate systems: a well-resourced private sector that serves the minority and the poorly resourced public sector that serves approximately 83% (50 million) of the population [5]. Individuals using the private health care system are either funded via medical aids/insurance or self-funded. Some individuals use both the private and public health care systems, using the latter when their medical aid funds have depleted or when they are facing monetary constraints [6].

The public health sector in South Africa is challenged with a multitude of illnesses including the HIV and tuberculosis epidemic and diseases of lifestyle [7]. Noncommunicable diseases are, however, a leading cause of death among adults in sub-Saharan Africa, which have a disproportionate impact on the economically active population [7].

Economic data from the United States indicate that the future cost implications of T1DM and its related emergency and long-term complications may have an unsustainable impact on patients and health care systems [8].

Epidemiological data on T1DM highlight a marked disparity in remaining life expectancy of people living with diabetes by income group. Although multifactorial, this disparity reinforces the need to improve access to diabetes education, blood glucose monitoring, skilled health care, and insulin [9]. Given the limited resources of the public health care system in South Africa and the large patient numbers under care, rational decision-making is required to optimally use limited human and financial resources.

The rising prevalence of T1DM, paralleled with the high mortality and morbidity of people living with diabetes, presents a challenge to health care systems and policy makers, particularly in the developing world [10]. Considering this, more effort should be channeled into reducing the financial burden on health care systems and patients [11]. To appropriately address the financial implications of managing people living with diabetes, pharmacoeconomic data are required. Unfortunately, to the best of our knowledge, there remain no data on the immediate and long-term costs of managing people with T1DM in the public health care setting.

It is through assessing and quantifying the health care resource utilization of patients living with T1DM that effective management initiatives can be implemented. Furthermore, this knowledge may help develop policies to ensure the optimal use of scarce resources.

### Rationale

The public health care sector has very limited data in general and specifically on people living with diabetes. Given that diabetes mellitus is one of the leading causes of death in South Africa, it is important to quantify and optimize resource utilization for patients with diabetes mellitus in the public health care sector. The resource utilization data generated from this study could assist with future policy development and optimization of care by considering the cost of illness, the cost of treatment, and optimal clinical outcomes.

### Definitions

An uncontrolled hemoglobin A_1c_ (HbA_1c_) reading will be defined as a reading ≥7%, and controlled T1DM will be defined as an HbA_1c_ reading <7% [12].

### Study Objectives

#### Primary Objective

The primary objective was to quantify the cost of health care resource utilization over a 12-month period in patients with controlled and uncontrolled T1DM in the public health care sector.

#### Secondary Objective

The secondary objective was to quantify the cost of health care resource utilization in patients with T1DM with controlled (HbA_1c_ <7%) versus uncontrolled (HbA_1c_ ≥7%) blood glucose level over a 12-month period and to compare the cost over a modeled 5-, 10-, and 25-year time horizon.

## Methods

### Study Design

This was a national, multicenter, and observational study where a representative sample of patients was observed. Data were collected retrospectively over a 12-month period preceding the date of informed consent from patient chart data in the South African public health care sector.

### Duration of Study Participation

A single visit was planned during which a patient signed an informed consent form to be included in the retrospective chart review. Eligibility criteria were confirmed and verified during the visit.

### Selection of Patients

Consecutive patients who fulfilled the eligibility criteria were enrolled at each site. Competitive recruitment was used for this study.

### Withdrawal Criteria

Withdrawal of informed consent was possible at the request of the patient or investigator.

### Sample Size

A sample population of 224 consenting patients with T1DM fulfilling the eligibility criteria was recruited to participate in the chart review. Patients were recruited from 2 sites.

### Determination of Sample Size

The sample size was calculated using SAS (version 9.4; SAS Institute) based on the assumption that both sites have a total population of 1129 patients with T1DM. The recommended HbA_1c_ readings were also taken into consideration; it was recommended that adults maintain an HbA_1c_ reading that is less than 7% to be classified as controlled T1DM. It was further assumed that 20% of the sample will have HbA_1c_ readings that are less than 7%. The population was also assumed to be normally distributed, using a margin of error of 5% and a confidence level of 95%; the sample size was calculated using the following formula:

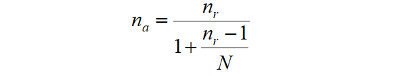



where *n_r_* is the required sample size, 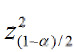 is the corresponding standard normal variate for 5% type 1 error, *P* is the proportion of the population whose HbA_1c_ readings are <7%, and *d* is the margin of error set to 0.05.

The result was further adjusted relative to the population size of 1129 patients:

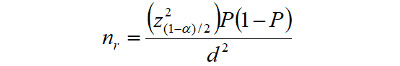



where *N* is the population size and *n_a_* is the adjusted sample size relative to the population size.

The total sample size was 224 patients, adjusted for a 10% rate of patient files that may not be evaluable because of missing or incomplete data.

The inclusion and exclusion criteria are given in Textbox 1.

**Inclusion and exclusion criteria.**

Inclusion criteria

- Signed informed consent at enrollment in the study
- Adults aged ≥18 years
- Patients with type 1 diabetes mellitus whose diagnosis had been confirmed
- History of being on human insulin therapy treatment for 12 months preceding the date of informed consent
- At least 1 hemoglobin A_1c_ reading recorded in the patient chart in the 12 months preceding the date of informed consent

Exclusion criteria

- Patients with type 2 diabetes mellitus
- Patients with gestational diabetes
- Patients with chronic pancreatitis
- Patients with latent autoimmune diabetes in adulthood
- Patients with maturity-onset diabetes in their youth
- Patients on analog insulin therapy (rapid acting and long acting)

### Modalities of Recruitment

Each investigator included consecutive subjects who met the eligibility criteria. This consecutive recruitment limited biases of subject selection. If the first patient did not fulfill the eligibility criteria or declined to participate, the investigator continued with the next patient until the enrollment target for the study was reached. A patient only signed the informed consent form once they were fully informed about the study and understood the information provided to them in the consent form. A patient-tracking log form was used to document enrollment and ensure anonymity. Travel reimbursement was allocated per patient in line with local regulations.

### Selection of Investigators

The investigators were chosen based on the following criteria: tertiary hospitals in South Africa and institutions with available comprehensive patient charts and records.

### Analysis Populations

The full analysis set consisted of all patients included in the study, meeting all inclusion criteria and not meeting any exclusion criteria or withdrawal criteria. The per-protocol set consisted of patients who met all inclusion criteria and had no major protocol violations.

### Statistical Methods

The data management team developed a statistical analysis plan before the database lock, which detailed the statistical analysis to be performed as well as the populations for analysis. A population review meeting was held after all data had been collected and cleaned and before the final database lock to determine which patients were analyzed in preparation for this retrospective analysis study.

Statistical analysis was performed after the database lock once all study data had been collected and cleaned. The statistical analysis was performed using SAS (version 9.4; SAS Institute).

### Analysis Variables

All the patients in the analysis population were included in the data set for analyses. The analysis of data collected in this study was mainly descriptive. All collected data and end point variables were summarized using descriptive statistics.

### Descriptive Analyses Included

For continuous variables, descriptive statistics included the number of patients, mean, SD, median, minimum, and maximum. Frequencies and percentages were displayed for categorical data. Percentages by categories were based on the number of patients with no missing data. Analysis was calculated according to the diagnosis group (uncontrolled vs controlled T1DM), as appropriate.

### Modeling

The IQVIA CORE Diabetes Model (CDM) will be used to evaluate the health care resource utilization over a 12-month period in patients with controlled and uncontrolled T1DM in the public health care sector. Furthermore, the model will be used to project 5-, 10-, and 25-year costs.

### Ethical Principles

This study was conducted in accordance with the principles laid by the 18th World Medical Assembly and all subsequent amendments [13]. Institutional permissions were received before the initiation of the study. Observation of both global and local regulations, including local data protection regulations, was maintained. Only data captured in patient records in the 12 months preceding the provision of informed consent were included in the study data. Participants were informed that their study-related data would be used by the sponsor in accordance with the local data protection law.

## Results

Ethics clearance (reference number 200407) and institutional permissions were acquired. Funding was awarded and enrollment began on February 9, 2021, and was completed on August 24, 2021, with 224 participants. A database lock was performed on October 29, 2021. The statistical analysis and clinical study report were completed in January 2022.

## Discussion

It is known that uncontrolled diabetes is associated with diabetic ketoacidosis and microvascular and macrovascular complications, which have associated cost implications. T1DM is a leading cause of morbidity, mortality, and health care resource utilization globally. Because the onset of T1DM typically arises early, effective disease management is imperative to limit its health and economic impact [14]. Existing literature has highlighted the higher cost of treating T1DM compared with T2DM [15]. To understand the economic impact of T1DM, the IQVIA CDM is used to evaluate the health care resource utilization over a 12-month period in patients with controlled and uncontrolled T1DM in the public health care sector. In addition, 5-, 10-, and 25-year projected costs are intended to be modeled to understand how costs change with the duration of the disease, assuming that patients maintain their current HbA_1c_ level.

Our discussion will largely focus on explaining the IQVIA CDM model, as it is a key instrument used to determine health resource utilization in this study.

The IQVIA CDM is a computer simulation model developed to determine the long-term health outcomes and economic consequence of interventions in T1DM and T2DM. The model is accessible on a licensed basis over the internet [16]. This is a non–product-specific diabetes analysis tool that performs real-time simulations taking into account intensive or conventional insulin therapy, oral antidiabetic drugs, screening and treatment strategies for microvascular complications, treatment strategies for end-stage complications, and multifactorial interventions [17].

The model simulates disease progression in both T1DM and T2DM. The IQVIA CDM is designed to take surrogate end points and translate them into long-term health economic outcomes. For example, HbA_1c_ is a surrogate marker that is linked to life expectancy [18].

Within the CDM, disease progression is based on a series of interdependent Markov submodels that simulate the progression of disease-related complications (angina, myocardial infarction, congestive heart failure, stroke, peripheral vascular disease, diabetic retinopathy, macular edema, cataract, hypoglycemia, ketoacidosis, nephropathy, end-stage renal disease, neuropathy, foot ulcer, and amputation) and other-cause mortality. The model is a fixed-time increment (annual) stochastic simulation with each submodel using time, state, and diabetes-type dependent probabilities. Monte Carlo simulations are performed at the individual patient level using tracker variables to accommodate complex interactions between submodels. The progression of relevant physiological parameters (eg, HbA_1c_, systolic blood pressure, lipids, and BMI) is simulated based on long-term epidemiological data, and event risk is constantly updated on the basis of these risk factors. The model facilitates interconnectivity and interaction between the modeled complications, representing the complex and varied sequelae of diabetes [19].

Analyses can be performed on patient cohorts, defined in terms of age, gender, baseline risk factors, and pre-existing complications. Economic and clinical data in the disease management module can be edited by the user, ensuring adaptability by allowing the inclusion of new data as they become available and facilitating the creation of country, health maintenance organization, or provider-specific versions of the model. In analyses, patients remain on initial treatment either for a set period of time or until a threshold of HbA_1c_ is reached; treatment duration and thresholds are set according to individual country practice [18].

A core outcome of the model is the estimate of the incremental cost per quality-adjusted life year gained. The model therefore requires the input of a comprehensive set of utility weightings for each model state. In version 9.0 of the CDM, default utilities are updated through the systematic review and subsequent assimilation of utility data into a utility data set consistent with the National Institute for Clinical Excellence reference case and applicable across multiple geographies [3]. Quality-of-life values are then calculated for every patient in each year of the simulation and used to estimate the average quality-adjusted life expectancy. Utilities are assessed on a scale from 0 to 1, where 0 represents death (no quality of life) and 1 indicates a healthy person without complications. In effect, for each acute event that occurs during 1 year of the simulation, a quality-of-life disutility value is used to adjust the overall quality-of-life utility value for the patient. Disutilities range from 1 to 0 and therefore cause the quality-of-life utility to either decrease or remain constant. After an event, patients change state, and the new state is associated with different state utilities. A minimum approach is applied to the estimation of utilities—in the case of multiple events, the lower utility is applied for that period. This is a simple and well-established approach to the application of utilities in the case of multiple interdependent health states [17,19].

Clinical and economic outcomes are calculated within the model using a nonparametric bootstrapping approach. This process simulates the lifetime progression of diabetes in a cohort of hypothetical patients repeating the process over numerous simulations. If 1000 patients are run through 1000 iterations, this produces 1000 mean values of clinical effectiveness and lifetime costs, which are then used to generate a scatter plot diagram and acceptability curves to express the likelihood of a treatment being cost-effective versus a comparator. In the base-case analyses, second-order uncertainty is not applied, and stability of outcomes is reached through a run of 1000 patients through 1000 iterations. There is an existing and emerging body of evidence looking to quantify health care resource utilization and appropriately describe economic considerations in the management of people living with T1DM [14,15]. There is, however, a lack of such data within developing nations that are required to facilitate policy discussions pertaining to the health and economic impact of T1DM in South Africa.

### Limitations

The study limitations include the retrospective study design and selection of only 2 health care facilities. Data entries were reliant on a chart review and hence entries from the treating health care worker.

### Conclusions

For chronic diseases such as T1DM, providing treatments and therapies that produce the best clinical outcomes while containing health care expenses is a matter of importance for providers, policy makers, and research at large. Quantifying the economic burden of T1DM is needed to effectively implement treatment strategies and policies that are cost-effective.

## Abbreviations

CDM: CORE Diabetes Model
HbA_1c_: hemoglobin A_1c_
T1DM: type 1 diabetes mellitus
T2DM: type 2 diabetes mellitus

## Data Availability

The data sets generated during this study are in various stages of manuscript publication and development, and thus are not currently available to the public.
